# Extrapancreatic Findings and Retrospective Diagnostic Pitfalls in Type 1 Autoimmune Pancreatitis: A Descriptive CT and MRI Study

**DOI:** 10.3390/diagnostics16193129

**Published:** 2026-09-26

**Authors:** Van Khang Le, Ton My Dieu Linh, Do Dang Tan, Dang Ngoc Hieu, Nguyen Thi Khoi, Nguyen Thi Thu Thao, Trinh Ha Chau, Cong Long Nguyen, Vu Dang Luu, Quang-Anh Nguyen

**Affiliations:** 1 Department of Radiology, Hanoi Medical University, Hanoi 100000, Vietnam; drkhang2006@gmail.com (V.K.L.); vudangluu@hmu.edu.vn (V.D.L.); 2 Institute of Diagnostic and Interventional Radiology, Bach Mai Hospital, Hanoi 100000, Vietnam; tonmydieulinh123@gmail.com (T.M.D.L.); tandaihocy@gmail.com (D.D.T.); hieuychmu@gmail.com (D.N.H.); nguyenthikhoi2903@gmail.com (N.T.K.); thuthaohmu@gmail.com (N.T.T.T.); chau.trinhha@gmail.com (T.H.C.); 3 Institute of Digestive Diseases, Bach Mai Hospital, Hanoi 100000, Vietnam; nguyenconglongbvbm@gmail.com

**Keywords:** autoimmune pancreatitis, immunoglobulin G4-related disease, magnetic resonance imaging, multidetector computed tomography, diagnostic errors, cholangiocarcinoma

## Abstract

**Background/Objectives:** Type 1 autoimmune pancreatitis (AIP) can resemble pancreatobiliary malignancy. We described pancreatic and extrapancreatic CT/MRI findings and explored retrospective interpretive patterns, including unrecognized diffuse disease. **Methods:** This retrospective single-center study included 40 selected patients with archived definite/probable JPS 2018 classifications. CT was available for 31 patients and MRI/MRCP for 36. Two radiologists reviewed features independently and reached consensus; access to diagnostic information during these assessments could not be reconstructed. Six operated patients formed an illustrative case series reviewed post hoc with final diagnoses known. **Results:** Diffuse disease occurred in 28/40 (70.0%), a capsule-like rim in 33/40 (82.5%), homogeneous portal venous and/or delayed enhancement in 40/40, and qualitative diffusion restriction in 36/36 MRI examinations. Extrapancreatic abnormalities compatible with IgG4-related involvement were recorded in 32/40 patients (80.0%): biliary abnormalities in 30/40 and renal abnormalities in 11/40. Two extrapancreatic presentations initiated evaluation. Three of six operated patients had diffuse disease. The narratives illustrate biliary-dominant findings, temporal change, and discordant multimodality evidence. These are selected-cohort frequencies, not population prevalence or sensitivity estimates. Imaging also contributed to diagnostic adjudication, creating incorporation bias; malignant controls were absent. **Conclusions:** The findings describe an imaging spectrum and generate educational hypotheses. The diagnostic-role framework has not been validated as a diagnostic algorithm or shown to reduce misdiagnosis or unnecessary surgery. Independent comparative validation is required.

## 1. Introduction

Type 1 autoimmune pancreatitis (AIP) is the pancreatic manifestation of immunoglobulin G4-related disease (IgG4-RD), a fibroinflammatory condition characterized by lymphoplasmacytic infiltration, storiform fibrosis, and obliterative phlebitis [1,2,3,4,5,6,7,8]. Although pancreatic enlargement, a capsule-like rim, delayed enhancement, and main pancreatic duct abnormalities are well-recognized imaging features, AIP may closely resemble pancreatic ductal adenocarcinoma (PDAC) or cholangiocarcinoma. The consequences of this overlap are substantial because AIP often responds to corticosteroid therapy, whereas suspected pancreatobiliary malignancy may lead to major surgery [9,10,11,12,13,14,15,16].

Previous imaging studies describe extrapancreatic involvement as supportive of AIP [5,17,18,19,20]. Its place in the diagnostic sequence is also clinically relevant: an extrapancreatic presentation may initiate investigation, while concurrent pancreatic, biliary, renal, salivary-gland, or retroperitoneal abnormalities may suggest a systemic process. Conversely, a prominent biliary abnormality may coexist with suspicion of malignancy. A multiorgan pattern should broaden the differential diagnosis rather than establish IgG4-RD or override evidence of cancer. These observations motivate an examination of interpretive patterns distinct from testing diagnostic accuracy.

The established discussion of diagnostic difficulty has also centered on focal or mass-forming AIP, particularly lesions in the pancreatic head that mimic PDAC [16,21,22,23,24,25,26,27,28,29,30]. Diffuse AIP is usually regarded as the more typical and recognizable morphology because diffuse enlargement, a capsule-like rim, long-segment ductal narrowing, and diffusion restriction may occur together [31,32,33,34,35]. However, this apparent diagnostic advantage assumes that the pancreas is actively assessed, prior examinations are available, and findings across modalities and organ systems are reconciled. When the clinical presentation is biliary or otherwise extrapancreatic, even diffuse disease may remain unrecognized.

Imaging data on type 1 AIP from Southeast Asia remain limited. We therefore described CT and MRI findings in a selected Vietnamese cohort of 40 patients with archived type 1 AIP diagnoses. A post hoc educational analysis examined extrapancreatic diagnostic-entry presentations and retrospective interpretive patterns in six patients who underwent surgery for suspected malignancy, including three with diffuse disease. The terms diagnostic-entry manifestation, pattern-completing clue, and dominant distractor organize the observations; they are not validated predictors, prospectively assigned outcomes, or established causes of diagnostic error. The study was not designed to estimate population prevalence, diagnostic performance, or prevention of surgery.

## 2. Materials and Methods

### 2.1. Study Design and Patients

This retrospective descriptive study examined a selected cohort evaluated at a single tertiary referral hospital in Vietnam between February 2020 and May 2026. Case ascertainment, data collection, and finalization of the analytic dataset were completed in May 2026. Clinical records, laboratory data, operative and histopathologic reports, and CT/MRI examinations stored in the picture archiving and communication system were reviewed. The retained register contained the final included cohort but not the total number initially screened or exclusions by reason (Supplementary Figure S1). Complete consecutive recruitment cannot therefore be verified. Final diagnoses were taken from archived multidisciplinary adjudications using the Japanese Clinical Diagnostic Criteria for Autoimmune Pancreatitis 2018 (JPS 2018) [31] rather than independently re-established for this analysis. The cohort represents patients already classified as having AIP, not an unselected population with an undiagnosed pancreatobiliary lesion.

Eligibility required an archived definite or probable type 1 AIP classification, diagnostic-quality pretreatment contrast-enhanced CT and/or pancreaticobiliary MRI, and sufficient retained records for descriptive review. Recorded exclusion criteria were incomplete or nondiagnostic imaging, unavailable essential records, pancreatobiliary malignancy that could not be reliably excluded, or only a possible JPS 2018 classification. JPS 2018 considers pancreatic enlargement, irregular main pancreatic duct (MPD) narrowing, serum IgG4 ≥ 135 mg/dL, histopathology, characteristic extrapancreatic lesions, and corticosteroid response [31]. The archived classifications comprised 33 definite and seven probable cases. A patient-level matrix of criterion combinations was not retained, precluding independent reconstruction (Supplementary Table S1). Histopathology contributed to adjudication in nine cases; imaging with elevated IgG4 was the principal recorded support in 23; and eight had composite support from imaging, IgG4, exclusion of malignancy, histopathology, and/or corticosteroid response. The contribution and timing of steroid response were not separately coded. JPS 2018 permits consideration of steroid response only after appropriate evaluation for malignancy, not as a substitute for that evaluation [31]; the retained data do not independently verify every case-level step. No formal sample-size calculation was performed; all eligible patients identified in the retained register were included.

Demographic data, presenting manifestations, serum IgG4, and extrapancreatic findings were extracted. All CT/MRI examinations used for the primary imaging analysis preceded corticosteroid treatment. IgG4 was available for 32 patients; its exact sampling date relative to imaging and treatment was unavailable for 31, whereas Case 06 had documented testing during the preoperative work-up. Values were summarized at 135 mg/dL and at the additional published threshold of 280 mg/dL [36], without treating either threshold as an independently validated test in this study or 280 mg/dL as the institutional assay upper limit. Pancreatic imaging and characteristic extrapancreatic lesions could contribute both to JPS adjudication and to the features described here. The reference diagnosis was therefore not independent of the imaging assessment, creating incorporation bias. Aggregate counts were not used to infer unrecorded patient-level criterion combinations.

### 2.2. Imaging Acquisition

Contrast-enhanced CT was performed on multidetector systems using a pancreatic protocol. Nonionic iodinated contrast material was administered intravenously at approximately 1.5–2 mL/kg, with an injection rate up to approximately 5 mL/s. Acquisitions included unenhanced, pancreatic/arterial (approximately 35–45 s), portal venous (60–70 s), and, when available, delayed (approximately 240 s) phases. Thin-section axial images and multiplanar reconstructions were evaluated.

Pancreaticobiliary MRI included precontrast T1-weighted imaging with or without fat suppression, T2-weighted imaging, magnetic resonance cholangiopancreatography (MRCP), diffusion-weighted imaging (DWI) with apparent diffusion coefficient (ADC) maps, and dynamic postcontrast T1-weighted imaging. Dynamic imaging generally included arterial, portal venous, and approximately 3 min delayed phases. Two-dimensional and/or three-dimensional MRCP was used to assess the pancreatic and biliary ducts. Twenty-seven patients underwent both CT and MRI, four underwent CT only, and nine underwent MRI only. DWI and ADC maps were available in all 36 MRI examinations. Exact b-values differed among scanners and were not consistently retrievable from the archived protocols; no quantitative ADC threshold was applied. Qualitative diffusion restriction was assessed on the highest available b-value image together with the corresponding visually low ADC signal. Acquisition parameters were not uniform because of the retrospective design, but all examinations included the sequences or phases needed for diagnostic review.

### 2.3. Image Analysis

Two radiologists with more than 5 years of hepatopancreatobiliary imaging experience retrospectively assessed the prespecified features independently. Discordant assessments were subsequently reviewed together until consensus; only the consensus dataset was retained. The records do not establish either reader’s access to clinical information, original reports, IgG4, or the final diagnosis during the initial readings or feature-level consensus. Neither blinding nor knowledge of the final diagnosis can therefore be assumed for either stage. Individual worksheets and scores were unavailable, so agreement, reader-specific frequencies, and the effect of consensus cannot be quantified. Consensus is a final classification, not a measure of reproducibility. This was not an independent blinded diagnostic-performance evaluation. Separately, consensus interpretation of the surgical narratives was undertaken post hoc with final AIP diagnoses and postoperative histopathology known (Section 2.4 and Section 2.5). Overall CT/MRI features used 40 patients; MRI-specific MPD and DWI findings used 36 examinations, comprising 25 diffuse and 11 non-diffuse cases.

Diffuse disease was defined as involvement of more than two-thirds of the pancreatic length, segmental disease as one-third to two-thirds, focal disease as less than one-third, and multifocal disease as two or more separate areas. Segmental, focal, and multifocal patterns were grouped as non-diffuse disease for descriptive comparison. Arterial hypoenhancement was defined as visually lower enhancement than uninvolved pancreatic parenchyma during the arterial phase. Dotted/speckled arterial enhancement was defined as multiple small enhancing foci within an otherwise relatively hypoenhancing-involved region. Homogeneous portal venous and/or delayed enhancement was recorded when the involved pancreas became relatively uniform in later phases. A capsule-like rim was a hypoattenuating CT or hypointense MRI band surrounding part or all of the involved pancreas. Qualitative diffusion restriction required a visually high signal on the highest available DWI b-value image with a corresponding low signal on the ADC map; no numeric ADC cut-off was used. The duct-penetrating sign indicated an MPD traversing a discrete lesion without abrupt complete interruption; the enhanced duct sign indicated enhancement of the MPD wall within the involved region.

### 2.4. Extrapancreatic Findings and Post Hoc Educational Framework

Extrapancreatic abnormalities compatible with IgG4-RD comprised biliary and renal findings, retroperitoneal fibrosis, and salivary-gland disease. A biliary abnormality compatible with IgG4-related involvement required intrinsic bile-duct wall thickening and/or enhancement or a segmental or multifocal stricture. Isolated upstream dilatation or smooth distal compression adjacent to an enlarged pancreatic head, without intrinsic mural abnormality, was considered a secondary obstruction and was insufficient for this classification. These operational criteria indicate imaging compatibility, not an organ-specific diagnosis. Histologic or dedicated cholangiographic confirmation was not uniform, and a separate count of independently confirmed IgG4-related sclerosing cholangitis (IgG4-SC) was unavailable. Renal abnormalities required characteristic cortical low-attenuation/low-enhancing round, wedge-shaped, or patchy lesions; renal pelvic soft-tissue thickening; or rim-like perirenal soft tissue. Simple cysts, chronic focal scars, and isolated nonspecific perinephric stranding were excluded. Retroperitoneal and salivary-gland findings were recorded from imaging and clinical records [17,18,19]. These definitions do not establish independent exclusion of inflammatory or malignant mimics for every extrapancreatic lesion.

The diagnostic-role framework was developed post hoc for educational description. A diagnostic-entry manifestation was an extrapancreatic presentation recorded as initiating the evaluation that identified pancreatic disease. A pattern-completing clue was an abnormality that could be interpreted retrospectively alongside pancreatic findings as a multiorgan pattern. A dominant distractor was a conspicuous abnormality emphasized in the preoperative malignant interpretation; the term does not establish a clinician’s cognitive process or a causal mechanism. Diagnostic-entry presentations were counted within the selected cohort. Pattern-completing and distractor labels were assigned qualitatively to the surgical narratives by consensus with final diagnoses and postoperative histopathology known. They were not mutually exclusive, independently reproducibility-tested, or prospectively defined diagnostic outcomes. No threshold, weighted score, or diagnostic algorithm was developed or validated.

### 2.5. Retrospective Review of the Illustrative Surgical Case Series

The six patients operated on for suspected malignancy formed an illustrative case series nested within the cohort, not a validation sample. Original diagnoses, procedures, pathology reports, and the retrievable sequence of CT, MRI/MRCP, endoscopic ultrasound (EUS), IgG4, and biopsy were reviewed (Supplementary Table S3). For this educational reappraisal, the radiologists reached consensus post hoc with the final AIP diagnoses and postoperative histopathology known; it was not a blinded rereading or prospective adjudication of diagnostic errors. Documented preoperative findings were distinguished from findings recognized on retrospective review and from postoperative evidence. Missing records were not interpreted as proof that an investigation or multidisciplinary discussion had not occurred. The review did not establish cognitive causes, isolate the effect of imaging from other clinical information, or determine whether any operation was avoidable. A cohort-wide inventory of earlier examinations, intervals, and decision-time image availability was not retained; the longitudinal observation in Case 05 is therefore illustrative.

Histopathology reports, rather than centrally re-reviewed slides, were used. Histopathology was considered to have contributed to adjudication when the report excluded malignancy and described chronic fibroinflammatory pancreatitis or pancreatobiliary inflammation considered compatible with AIP/IgG4-RD. Detailed JPS histology components—including IgG4-positive cells per high-power field, storiform fibrosis, and obliterative phlebitis—were not uniformly reported or retained, and histologic JPS subcategories could not be reconstructed retrospectively. Supplementary Table S2 distinguishes the verified report-level findings from features that were not retrievable for all nine pathology-supported cases.

### 2.6. Statistical Analysis

SPSS version 20.0 was used. Categorical variables are reported as numbers and percentages and continuous variables as the mean ± standard deviation and range. All frequencies are conditional on inclusion in this selected cohort and availability of the relevant examination; they are not population prevalence or sensitivity estimates. The study lacked a representative diagnostic-evaluation sample, an independent reference standard, and PDAC/cholangiocarcinoma controls. No diagnostic-performance estimates, inferential group comparisons, or predictive models were undertaken. Missing data were not imputed; available-case denominators are reported for each variable. The six-case narrative analysis was qualitative and did not measure effects on misdiagnosis or surgery.

## 3. Results

### 3.1. Cohort Characteristics

The source register did not retain the total number initially screened or exclusions by reason; all verifiable flow counts are shown in Supplementary Figure S1. The final cohort contained 40 patients (mean age, 66.2 ± 9.9 years; range, 44–85 years), of whom 34 (85.0%) were men. The archived JPS 2018 classification was definite in 33 and probable in seven. Twenty-seven patients had both CT and MRI, four had CT only, and nine had MRI only. Obstructive jaundice was present in 28 (70.0%) and abdominal pain in 24 (60.0%). Serum IgG4 was measured in 32 patients; all exceeded 135 mg/dL, and 26/32 (81.3%) exceeded 280 mg/dL. Except for preoperative testing in Case 06, exact sampling dates relative to imaging and treatment were not retained. The 280 mg/dL threshold was used only as a published higher-specificity cut-off and not as the institutional assay reference limit. Aggregate diagnostic evidence and its availability are summarized in Supplementary Table S1. Six patients (15.0%) had undergone surgery because pancreatobiliary malignancy was suspected (Table 1).

### 3.2. Pancreatic CT and MRI Findings

Diffuse disease was recorded in 28/40 patients (70.0%). The 12 non-diffuse cases comprised four focal, four segmental, and four multifocal patterns; all involved the pancreatic head. Enlargement at the involved site occurred in all diffuse cases and 9/12 non-diffuse cases. A capsule-like rim was recorded in 33/40 (82.5%), and homogeneous portal venous and/or delayed enhancement in 40/40 (100.0%). The latter pools available later phases rather than a uniform delayed-phase protocol. Dotted/speckled arterial enhancement occurred in 10/28 diffuse and 9/12 non-diffuse cases (Table 2). These descriptive observations are conditioned by case selection, incorporation of imaging into adjudication, and retrospective consensus assessment; they do not establish sensitivity or discriminatory value.

Among 36 patients with MRI/MRCP, MPD narrowing was recorded in 27 (75.0%). All 36 examinations were scored as showing qualitative diffusion restriction on the highest available b-value image with a corresponding low ADC signal. The 36/36 count is retained as an observed consensus finding, not a standardized quantitative DWI result or a sensitivity estimate. In the 11 non-diffuse cases with MRI/MRCP, the duct-penetrating sign was recorded in seven (63.6%) and the enhanced duct sign in eight (72.7%). No ability to distinguish these findings from malignancy was tested.

### 3.3. Extrapancreatic Findings and Recorded Diagnostic-Entry Presentations

Extrapancreatic abnormalities compatible with IgG4-RD were recorded in 32/40 patients (80.0%). Biliary abnormalities meeting the operational definition occurred in 30/40 (75.0%), renal abnormalities in 11/40 (27.5%), retroperitoneal fibrosis in 4/40 (10.0%), and salivary-gland findings in 1/40 (2.5%). Eleven patients had compatible abnormalities involving at least two extrapancreatic organs, and nine had a pancreatic–biliary–renal pattern (Table 3). The 30 biliary observations are not 30 independently confirmed IgG4-SC diagnoses; that confirmed-disease count could not be determined.

Two patients had recorded extrapancreatic presentations that initiated evaluation: bilateral submandibular-gland enlargement in one and urinary obstruction caused by retroperitoneal fibrosis in the other. Neither had pancreas-related presenting symptoms. Contrast-enhanced abdominal CT during this evaluation disclosed pancreatic abnormalities and prompted an integrated work-up for IgG4-RD. These are descriptions of two recorded pathways, not estimates of the yield of screening patients with extrapancreatic disease. Pattern-completing clues and dominant distractors refer to the qualitative retrospective interpretation of the surgical narratives; a cohort-wide frequency or independently measured change in diagnostic confidence was not available for these labels.

### 3.4. Illustrative Surgical Case Series: Retrospective Interpretive Patterns

Six patients, aged 56–74 years, underwent pancreatobiliary surgery after an initial diagnosis suspicious for malignancy. All had jaundice and abdominal pain and had undergone both CT and MRI; only Case 06 had serum IgG4 documented before surgery. Postoperative histopathology excluded malignancy in all six and was recorded as compatible with type 1 AIP/IgG4-RD. For Cases 01–05, the retained analytic record did not preserve component-level histologic findings or IgG4 immunostaining counts. For Case 06, the report documented chronic fibrosing inflammation of the common bile duct and chronic pancreatitis compatible with IgG4-RD, together with chronic active perforated gastroduodenal ulceration and Candida in necrotic tissue. Three additional non-surgical cases had histopathology recorded as contributing to diagnostic adjudication, but their specimen-level details were not retained in the analytic dataset. The verified histopathologic evidence and these limitations are shown in Supplementary Table S2. Three surgical patients had non-diffuse disease and three had diffuse disease (Table 4).

Supplementary Table S3 distinguishes the retained chronology from retrospective interpretation. Cases 01–02 had focal head lesions and an initial tumor diagnosis; the archived table reports no preoperative IgG4 testing. Case 03 had a head–body lesion, arterial hypoenhancement, venous narrowing, MPD dilatation, and upstream atrophy. Case 04 had diffuse enlargement, an intrinsic distal bile-duct abnormality prominent in the original diagnosis, and bilateral renal lesions. In Case 05, earlier diffuse enlargement was followed approximately six months later by atrophy and prominent hilar disease. The availability of earlier images to the treating team was not documented. Case 06 had discordant CT, MRI, EUS, biopsy, and IgG4 findings. Exact dates for several events and the timing of communication of results were unavailable.

In Cases 01–02, retrospective review identified speckled/progressive enhancement, a capsule-like rim, and pancreatic duct abnormalities in focal head lesions originally interpreted as tumors. A renal abnormality was also described in one of these two patients (Figure 1). Individual case linkage was inconsistent between the retained narrative and figure records, so that renal observation is reported jointly rather than reassigned (Supplementary Table S3). The third non-diffuse case had an appearance resembling locally advanced PDAC; homogeneous later-phase enhancement, a rim, and a biliary abnormality supplied an alternative retrospective interpretation, not proof of benignity before surgery.

The three diffuse surgical cases illustrated different retrospective patterns. In Case 04, the preoperative diagnosis emphasized a distal bile-duct lesion despite concurrent diffuse pancreatic and renal abnormalities (Figure 2). In Case 05, earlier diffuse enlargement and subsequent atrophy accompanied a later diagnosis of Klatskin tumor type IV (Figure 3). This paired observation cannot establish whether earlier imaging was reviewed or whether comparison would have changed management. Case 06 illustrates multimodality discordance (Figure 4). None of these narratives quantifies the frequency or establishes the cause of diagnostic error.

In Case 06, EUS described a 2.4 × 2.5 cm distal common bile-duct lesion with suspected duodenal invasion, while CT and MRI suggested different pancreatic and biliary interpretations. A duodenal-ulcer mucosal biopsy showed chronic inflammation without malignant cells; a representative EUS-guided core of the pancreatic or bile-duct lesion was not documented. Serum IgG4 was recorded as 897 mg/dL during the preoperative work-up, but the assay reference interval and exact timing relative to imaging, communication of the result, and the operative decision were unavailable. Preoperative documentation therefore does not establish decision-time actionability. After pancreatoduodenectomy, histopathology showed chronic fibrosing common bile-duct inflammation and chronic pancreatitis compatible with IgG4-RD, active perforated gastroduodenal ulceration, and Candida in necrotic tissue. These postoperative findings support only an inferential inflammatory explanation for the EUS impression; they were not evidence available to the original decision-makers. Table 5 summarizes the retrospective patterns and their interpretive limits.

## 4. Discussion

### 4.1. Principal Findings

This selected cohort documents an imaging spectrum and six illustrative surgical narratives. Extrapancreatic abnormalities were recorded in 32/40 patients, and two extrapancreatic presentations initiated investigation. Three operated patients had diffuse disease, showing that an appearance considered characteristic in retrospect can coexist with a preoperative malignant interpretation. The contribution is educational: it distinguishes the presence of imaging findings from their recognition, temporal availability, and integration with other evidence. Selection, incorporation, possible review bias, and absent malignant controls prevent independent diagnostic or causal inference.

### 4.2. Extrapancreatic Abnormalities: Diagnostic Context and Limits of Confirmation

The 32/40 frequency describes the retained referred cohort, not the prevalence of extrapancreatic disease among all patients with AIP. Published studies provide context for biliary and renal manifestations [5,17,18,19,20,37,38], but agreement with those reports would not remove selection bias. The two submandibular/retroperitoneal presentations illustrate that evaluation can begin without pancreatic symptoms. They do not measure the yield or outcomes of routinely investigating pancreatic disease in an unselected extrapancreatic population.

The pancreatic–biliary–renal pattern in nine patients is co-occurrence, not a validated predictor combination. Pancreatic and characteristic extrapancreatic findings can contribute to JPS classification; reporting them again in cases selected through that process is subject to incorporation bias, not independent confirmation. Moreover, the operational biliary definition indicates compatibility, not definitive IgG4-SC. Bile-duct thickening or stricturing requires differentiation from malignancy and other inflammatory cholangiopathies using the clinical setting, serology, and appropriate tissue evaluation [39]. Isolated obstruction from pancreatic enlargement is a separate phenomenon. A confirmed IgG4-SC denominator was unavailable, and individual renal abnormalities were not uniformly independently confirmed as IgG4-related disease.

In the surgical narratives, prominent distal or hilar biliary findings coexisted with suspicion of cancer despite abnormalities retrospectively compatible with a systemic process. Dominant distractor describes that apparent emphasis, not a measured cognitive error; pattern-completing clue denotes retrospective coherence, not a demonstrated change in diagnostic confidence. A multiorgan interpretation can broaden the differential diagnosis but should not automatically supersede convincing malignant evidence. Its incremental value and risk of false-positive AIP attribution require evaluation in comparable pancreatic-mass and biliary-stricture populations.

### 4.3. Diffuse Disease, High-Frequency Signs, and Longitudinal Interpretation

Focal AIP is a recognized mimic of PDAC in the literature, particularly with a head pseudomass, obstructive jaundice, hypoenhancement, vascular narrowing, or upstream ductal change [16,21,22,23,24,25,26,27,28,29,30,40,41]. The non-diffuse cases illustrate this overlap. Three of the six operated patients had diffuse disease, despite its relatively characteristic description in diagnostic criteria and the imaging literature [31,32,33,34,35]. This observation does not estimate or compare misdiagnosis risk by morphology: no standardized diagnostic-error outcome was assessed across all diffuse and non-diffuse patients.

The 36/36 qualitative diffusion-restriction observation warrants particular caution. The denominator consists of available MRI examinations, not all 40 patients or an unselected diagnostic population. Assessment used heterogeneous, incompletely retrievable b-values and a visually low ADC signal without a numerical threshold. Thus, 36/36 denotes a retrospective consensus observation in selected patients with archived AIP diagnoses; it is neither a sensitivity estimate nor evidence of differentiation from PDAC or cholangiocarcinoma. Selection, incorporation, and possible observer or confirmation bias may contribute, but their separate effects cannot be quantified. Universal positivity cannot be attributed to selection alone, and no transferable ADC cut-off or cancer-exclusion rule can be derived.

The same distinction applies to the capsule-like rim (33/40; 82.5%) and homogeneous portal venous and/or delayed enhancement (40/40; 100.0%). The enhancement variable pools available later phases rather than one standardized delayed-phase test. These are descriptive frequencies, not sensitivity estimates, and cannot rank individual or combined signs by discriminatory performance.

Case 05 provides a single longitudinal illustration: earlier diffuse enlargement was followed by atrophy, while hilar biliary disease became prominent. Prior studies describe evolution toward chronic pancreatitis-like change [42,43], providing context rather than proof of mechanism or untreated natural history in this patient. Without a cohort-wide inventory of earlier imaging and its decision-time availability, we cannot determine how often such trajectories occurred or whether comparison would have changed treatment. In Case 06, IgG4, recorded as 897 mg/dL, increased the plausibility of an inflammatory explanation but did not independently exclude malignancy [3,14,36,44,45]. Its communication and weighting in the operative decision cannot be reconstructed. Postoperative ulceration and Candida must not be treated as missed preoperative evidence.

### 4.4. Retrospective Interpretive Considerations

Table 5 presents retrospective interpretive considerations, not corrective interventions or a decision rule. Reviewing multiphasic enhancement, ductal morphology, extrapancreatic abnormalities, and available earlier images is consistent with existing guidance [3,14,31,32,33,34,35]. These cases illustrate why those comparisons may be relevant but provide no tested probability threshold, minimum number of signs, or mandatory management pathway. Neither benign resection histology nor a plausible alternative interpretation proves that an operation was unnecessary given contemporaneous clinical uncertainty.

Further investigation when AIP and malignancy remain competing diagnoses must consider the clinical context, biopsy target and adequacy, and established diagnostic guidance rather than this educational framework alone [3,14]. A corticosteroid trial is not a substitute for appropriate malignancy evaluation. These considerations do not mandate biopsy in every resectable cancer or justify delaying a convincing oncologic diagnosis. Their effect on diagnostic accuracy, delay, and surgery was not measured in this cohort.

### 4.5. EUS and EUS-Guided Tissue Acquisition

The following discussion is drawn from published evidence, not a pathway tested in our cohort. EUS can characterize pancreatic parenchyma, ducts, and suspected lesions and enable targeted tissue acquisition, but a morphological impression of a mass or invasion is not specific for AIP [14]. When pancreatic tissue acquisition is indicated, EUS-guided fine-needle biopsy (EUS-FNB) can provide core architecture and immunostaining, whereas fine-needle aspiration (FNA) primarily samples cytology [46]. Preserved architecture permits assessment for lymphoplasmacytic sclerosing pancreatitis (LPSP), including storiform fibrosis and obliterative phlebitis; increased IgG4-positive cells alone are insufficient [2,14].

Two AIP-specific prospective studies support the feasibility of informative core sampling. The randomized COMPAS study compared a 22-gauge Franseen-tip with a 20-gauge forward-bevel FNB needle, finding a higher level 1/2 LPSP yield with the former; Ishikawa et al. also reported useful histologic yield with a 22-gauge Franseen needle [47,48]. These studies support histologic assessment, not guaranteed exclusion of cancer or avoidance of surgery.

Mangiavillano et al. compared EUS-FNB with macroscopic on-site evaluation (MOSE) against a fixed three-pass approach in pancreatic and nonpancreatic solid lesions [49]. MOSE-guided sampling had noninferior diagnostic accuracy with fewer passes. This informs sampling practice and specimen assessment; it did not validate an AIP-specific algorithm or demonstrate reduced surgery in AIP.

Case 06 illustrates the need to match tissue to the lesion of concern. The nonmalignant biopsy sampled a duodenal mucosal ulcer, not a documented pancreatic or distal bile-duct core; it could not exclude a deeper unsampled malignancy or establish pancreatic LPSP. A negative or nondiagnostic result requires review of target representativeness, specimen adequacy, and imaging–pathology concordance. Persistent suspicion may require repeat or alternative location-appropriate sampling [14,50]. Biliary-stricture sampling differs from pancreatic-mass FNB; potentially curable hilar lesions require specialist planning of the route and oncologic options [50]. No benefit of an unperformed or undocumented FNB procedure can be assigned retrospectively to Case 06.

### 4.6. Priorities for Prospective Validation

Future evaluations should consecutively enroll patients with indeterminate pancreatic masses, biliary strictures, or suspected AIP before the final diagnosis, retaining screening and exclusion logs. PDAC, cholangiocarcinoma, and relevant benign inflammatory mimics should be represented. Standardized CT/MRI, prespecified features and ADC methods, independent reader scores, and staged assessments of imaging with and without serologic/tissue information would permit reproducibility and incremental-value analyses. An independent reference panel should not use the proposed index score to determine the diagnosis; residual dependence arising from imaging-containing AIP criteria would still require explicit assessment.

Patient-level criterion combinations, IgG4 timing and assay limits, biopsy targets and adequacy, prior-image availability, and decision-time evidence should be retained. Independent external evaluation should measure both diagnostic performance and management outcomes, including missed or delayed cancer, benign resection, repeat-procedure burden, and treatment delay. A reduction in benign surgery would indicate benefit only without increased cancer-related harm. These are priorities for future evaluations, not a protocol implemented or validated in the present study.

### 4.7. Limitations

This study comprises 40 selected patients with archived AIP diagnoses at one tertiary center. Missing screening and exclusion counts prevent verification of complete consecutive recruitment or quantification of referral, selection, and spectrum bias. Frequencies should not be generalized to non-referral populations or undiagnosed pancreatobiliary lesions. JPS adjudication incorporated features also described in the analysis, creating incorporation bias. Patient-level criterion combinations were unavailable, and the nine pathology-supported cases do not supply a uniformly documented independent reference standard. There were no malignant controls or diagnostic/management intervention outcomes.

Exact IgG4 sampling dates were unavailable for 31/32 tested patients, and steroid-response contribution and timing were not separately coded. Histopathology relied on reports without central slide rereview; detailed IgG4 cell counts, storiform fibrosis, and obliterative phlebitis were not uniformly retrievable. CT/MRI availability and protocols differed, DWI b-values were incompletely retained, and no quantitative ADC threshold was used. Organ-specific confirmation of extrapancreatic abnormalities was not uniform. A cohort-wide denominator of earlier examinations was unavailable. Inconsistent linkage of the focal-head renal example prevented assignment to Case 01 or 02; this uncertainty is retained explicitly rather than resolved by inference.

For initial image readings and feature-level consensus, access to clinical and final diagnostic information cannot be reconstructed. Individual scores were unavailable, so consensus cannot establish reproducibility or correct possible confirmation bias. In contrast, the surgical narrative reappraisal was explicitly outcome-informed and susceptible to hindsight and observer bias. The post hoc labels were not reproducibility-tested or compared with prospective cognitive or management outcomes. The six cases are illustrative; neither causal mechanisms of misdiagnosis nor reductions in unnecessary surgery have been established.

## 5. Conclusions

In this selected retrospective cohort, extrapancreatic abnormalities compatible with IgG4-RD were recorded in 32/40 patients, and two extrapancreatic presentations initiated evaluation. Three of six operated patients had diffuse disease. The surgical narratives provide educational illustrations of biliary-dominant findings, temporal change, and multimodality discordance. These observations are subject to selection, incorporation, and possible review bias and do not establish population prevalence or diagnostic performance. The proposed diagnostic-role framework and retrospective interpretive considerations have not been validated as a diagnostic algorithm and have not been shown to reduce image misinterpretation, misdiagnosis, or unnecessary surgery. Comparative validation with independent assessment and prespecified diagnostic and management outcomes is required.

## Figures and Tables

**Figure 1:**
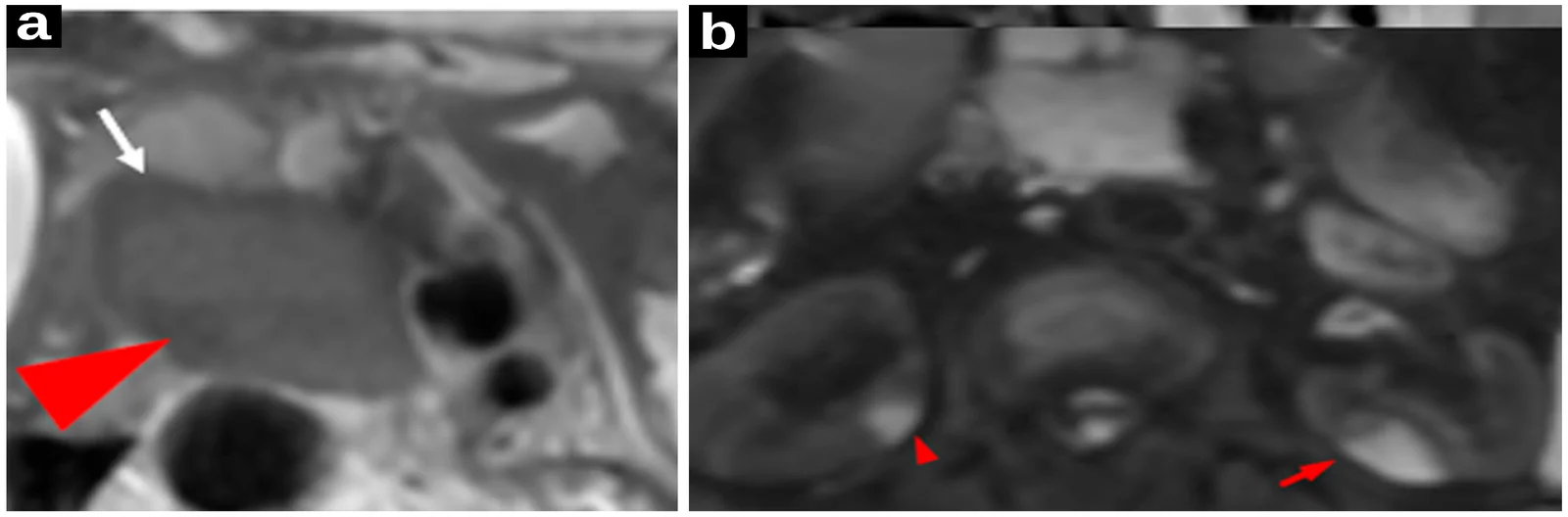
Illustrative focal pancreatic-head and renal abnormalities on MRI in a patient with an archived type 1 AIP diagnosis. (**a**) T2-weighted MRI shows focal head enlargement; the red arrowhead marks the hypointense capsule-like rim, and the white arrow marks biliary dilatation. (**b**) Contrast-enhanced MRI shows bilateral renal lesions (red arrowhead and arrow). Individual case numbering was not assigned because the retained figure and narrative records have inconsistent linkage; this limitation is documented in Supplementary Table S3.

**Figure 2:**
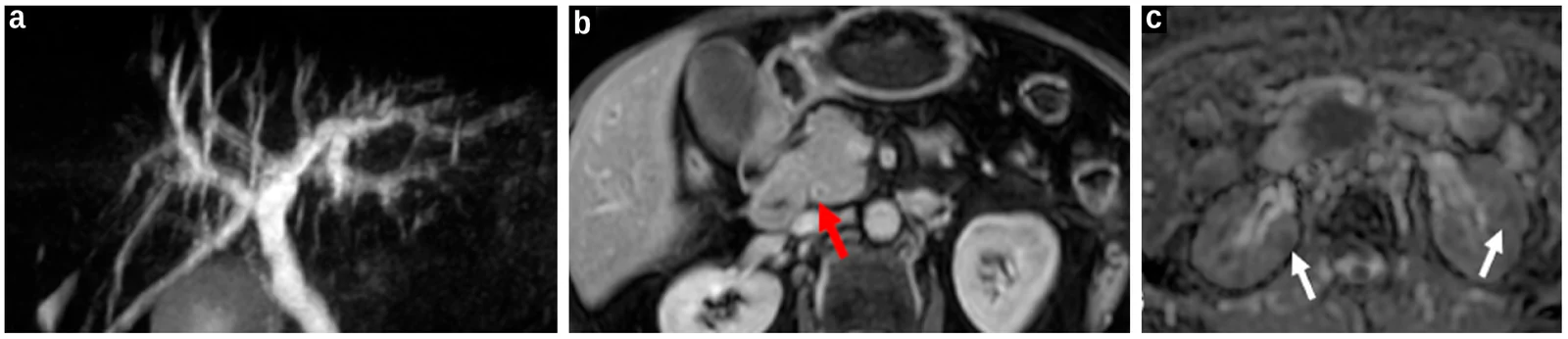
Diffuse pancreatic and extrapancreatic abnormalities in a 68-year-old woman (Case 04). (**a**) MRCP shows intrahepatic and extrahepatic biliary dilatation. (**b**) Axial MRI shows diffuse pancreatic enlargement and a capsule-like rim (red arrow). (**c**) Postcontrast MRI shows bilateral renal lesions (white arrows). The multiorgan pattern is compatible with IgG4-RD; these images alone do not establish IgG4-SC or exclude a malignant biliary lesion.

**Figure 3:**
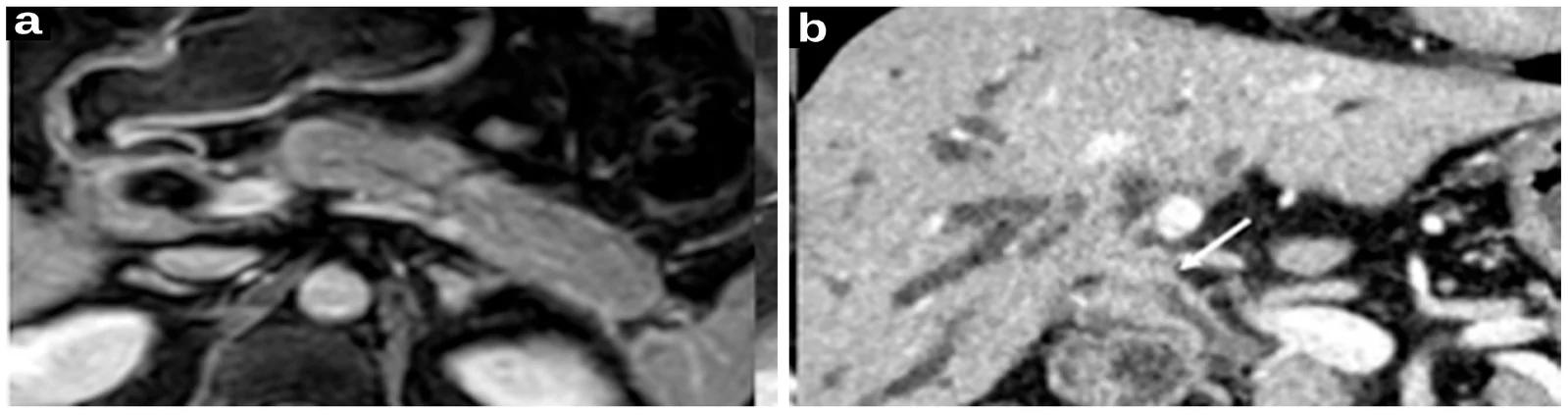
Illustrative temporal change in Case 05, a 74-year-old man. (**a**) Earlier MRI shows diffuse pancreatic enlargement with bile-duct wall thickening. (**b**) CT approximately six months later shows pancreatic atrophy and prominent hilar biliary abnormality (white arrow). This single observation does not establish a progression frequency, untreated natural history, or the effect of longitudinal comparison on management. Decision-time availability of the earlier images was not documented.

**Figure 4:**
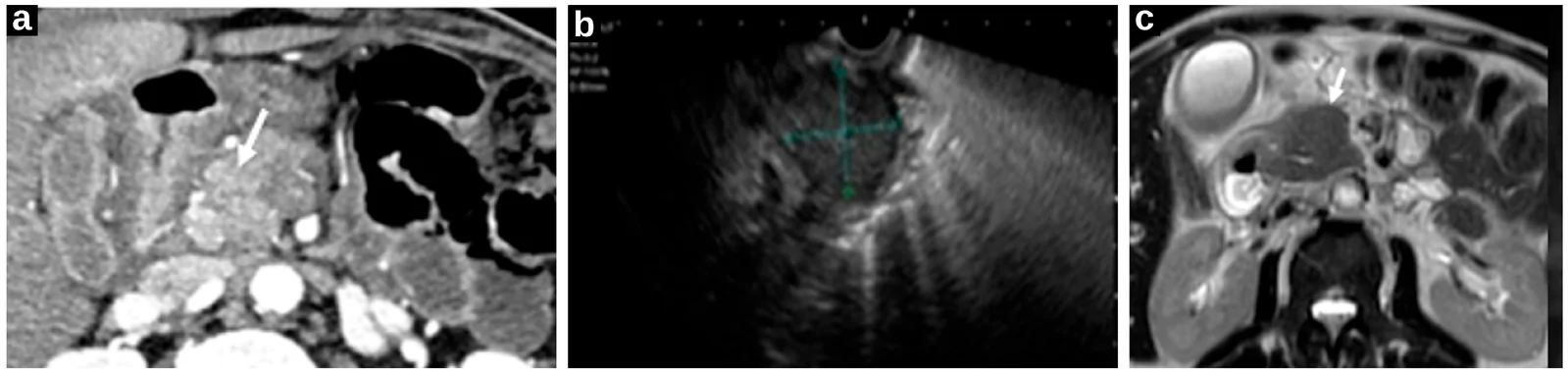
Discordant multimodality findings in a 65-year-old man with an archived diagnosis of diffuse type 1 AIP and preoperative serum IgG4 recorded as 897 mg/dL. (**a**) CT shows dotted/speckled enhancement in the pancreatic head against relative hypoenhancement of the body and tail (white arrow). (**b**) EUS calipers outline a 2.4 × 2.5 cm distal common bile-duct lesion reported as suspicious for duodenal invasion. The nonmalignant biopsy was from a duodenal mucosal ulcer, not a documented EUS-guided core sample of the pancreatic/biliary lesion; it cannot establish the nature of that deeper lesion. (**c**) MRI shows diffuse pancreatic involvement with a capsule-like rim (white arrow).

**Table 1:** Clinical, imaging, and diagnostic characteristics of the cohort.

Characteristic	*n* (%)/Mean ± Standard Deviation (Range)
Age, years	66.2 ± 9.9 (44–85)
Male sex	34 (85.0)
Obstructive jaundice	28 (70.0)
Abdominal pain	24 (60.0)
Acute pancreatitis	5 (12.5)
Weight loss	5 (12.5)
Contrast-enhanced CT available	31 (77.5)
MRI/MRCP available	36 (90.0)
Archived JPS 2018 classification: definite type 1 AIP	33 (82.5)
Archived JPS 2018 classification: probable type 1 AIP	7 (17.5)
Serum IgG4 measured	32 (80.0)
Serum IgG4 >135 mg/dL among tested patients	32/32 (100.0)
Serum IgG4 >280 mg/dL among tested patients	26/32 (81.3)
Extrapancreatic abnormalities compatible with IgG4-RD	32 (80.0)
Surgery because malignancy was suspected	6 (15.0)

Data are numbers (percentages) or means ± standard deviation (range) within the selected cohort. JPS categories are archived classifications, not independently re-established diagnoses. Extrapancreatic abnormalities indicate compatibility with IgG4-RD, not uniform organ-specific confirmation. The 280 mg/dL threshold is a published descriptive cut-off, not the institutional assay upper limit. AIP, autoimmune pancreatitis; JPS, Japan Pancreas Society; MRCP, magnetic resonance cholangiopancreatography.

**Table 2:** Descriptive within-cohort frequencies of CT and MRI findings by pancreatic morphology.

Imaging Feature	Diffuse Disease (n = 28)	Non-Diffuse Disease (n = 12)
Pancreatic enlargement at involved site	28 (100.0)	9 (75.0)
Capsule-like rim	24 (85.7)	9 (75.0)
Arterial hypoenhancement	6 (21.4)	5 (41.7)
Dotted/speckled arterial enhancement	10 (35.7)	9 (75.0)
Homogeneous portal venous and/or delayed enhancement	28 (100.0)	12 (100.0)
MPD narrowing on MRI/MRCP	18/25 (72.0)	9/11 (81.8)
Duct-penetrating sign on MRI/MRCP	Not assessed	7/11 (63.6)
Enhanced duct sign on MRI/MRCP	9/25 (36.0)	8/11 (72.7)
Qualitative diffusion restriction on DWI/ADC	25/25 (100.0)	11/11 (100.0)
Extrapancreatic abnormalities compatible with IgG4-RD	24 (85.7)	8 (66.7)

Data are numbers (percentages) within the selected cohort, not diagnostic-performance estimates. Overall CT/MRI variables use 40 patients; MRI/MRCP denominators are 25 diffuse and 11 non-diffuse cases. The duct-penetrating sign was assessed only for discrete non-diffuse lesions. Homogeneous enhancement combines available portal venous and/or delayed phases. DWI used heterogeneous b-values and no numerical ADC threshold. Extrapancreatic abnormalities indicate imaging/record-based compatibility, not uniform organ-specific confirmation. ADC, apparent diffusion coefficient; DWI, diffusion-weighted imaging; MPD, main pancreatic duct; MRCP, magnetic resonance cholangiopancreatography.

**Table 3:** Extrapancreatic abnormalities compatible with IgG4-RD and recorded diagnostic-entry presentations (n = 40).

Finding or Diagnostic Role	Patients	Percentage
Any extrapancreatic abnormality compatible with IgG4-RD	32	80.0
Biliary abnormality compatible with IgG4-related involvement	30	75.0
Renal abnormalities compatible with IgG4-RD	11	27.5
Retroperitoneal fibrosis	4	10.0
Salivary-gland involvement	1	2.5
At least two extrapancreatic organs with compatible abnormalities	11	27.5
Combined pancreatic–biliary–renal pattern	9	22.5
Extrapancreatic manifestation as diagnostic entry point	2	5.0

Categories may overlap; all percentages use the selected cohort of 40 patients. Biliary abnormalities required intrinsic mural change or segmental/multifocal stricturing rather than isolated secondary obstruction. Compatible biliary and renal findings were not uniformly independently confirmed as organ-specific IgG4-RD. Only diagnostic-entry presentations were counted; pattern-completing clue and dominant distractor are qualitative, unvalidated educational labels.

**Table 4:** Patients who underwent surgery because malignancy was suspected.

Case	Age/Sex and Morphology	Preoperative Serum IgG4	Initial Diagnosis	Operation
01	56/M; focal pancreatic head	Reported not performed	Pancreatic head tumor	Pancreatoduodenectomy
02	62/F; focal pancreatic head	Reported not performed	Pancreatic head tumor	Pancreatoduodenectomy
03	70/M; focal head-body lesion	Reported not performed	Pancreatic head-body tumor	Extended pancreatoduodenectomy
04	68/F; diffuse	Reported not performed	Distal common bile-duct tumor	Pancreatoduodenectomy
05	74/M; diffuse, evolving to atrophy	Reported not performed	Klatskin tumor type IV	Biliary surgery/segmental hepatectomy
06	65/M; diffuse	897 mg/dL (preoperative)	Pancreatic tumor or distal bile-duct tumor invading duodenum	Pancreatoduodenectomy

The archived summary reports preoperative IgG4 as not performed in Cases 01–05; this is retained as a report-level statement rather than an independent audit of laboratory ordering. Postoperative pathology did not show malignancy and contributed to AIP/IgG4-RD adjudication, without uniform retained component-level histology. Supplementary Tables S2 and S3 give the available evidence and chronology. AIP, autoimmune pancreatitis; F, female; M, male.

**Table 5:** Illustrative post hoc interpretive patterns in the six surgical cases.

Illustrative Pattern (Case)	Findings Associated with Preoperative Suspicion	Findings Recognized on Retrospective Review	Retrospective Interpretive Consideration
Focal pancreatic-head pseudomass (01–02)	Pancreatic-head lesion with biliary obstruction	Speckled/progressive later-phase enhancement; capsule-like rim; MPD abnormalities; compatible biliary findings; renal abnormality in one patient, with case linkage unresolved	An alternative multiorgan interpretation is educational, not a tested predictor combination. Archived IgG4 testing was reported as not performed; preoperative biopsy details were unavailable.
Appearance resembling locally advanced PDAC (03)	Arterial hypoenhancement, venous narrowing, MPD dilatation, and upstream atrophy	Homogeneous later-phase enhancement; capsule-like rim; compatible biliary abnormality	The signs coexisted with an archived nonmalignant diagnosis. Neither specificity nor the avoidability of surgery can be established from this case.
Biliary-dominant diffuse disease (04)	Distal bile-duct thickening/stricture prominent in the preoperative tumor diagnosis	Diffuse pancreatic enlargement and bilateral renal lesions accompanying the intrinsic biliary abnormality	The multiorgan pattern permits retrospective consideration of IgG4-RD but does not establish IgG4-SC, exclude cancer, or measure a cognitive cause of the original interpretation.
Illustrative temporal change (05)	Prominent hilar biliary disease after pancreatic atrophy developed	Earlier diffuse enlargement followed approximately six months later by atrophy	Earlier-image availability to the treating team was undocumented. One case cannot quantify a trajectory or establish that comparison would have changed management.
Multimodality discordance (06)	Head-predominant CT abnormality; EUS-reported 2.4 × 2.5 cm distal bile-duct lesion with suspected duodenal invasion	Diffuse pancreatic findings, speckled enhancement, and ductal abnormalities. IgG4 897 mg/dL and a nonmalignant mucosal-ulcer biopsy were documented preoperatively, but decision-time availability was uncertain.	A negative mucosal-ulcer biopsy is not representative sampling of the deeper lesion. Postoperative inflammation/Candida was not preoperative evidence; benefits from targeted FNB or alternative management were not tested.

This post hoc educational framework summarizes consensus interpretations made with final diagnoses and postoperative pathology known. Retrospectively visible features are not equivalent to documented recognition before surgery. Reproducibility has not been assessed; the framework has not been validated as a diagnostic algorithm or shown to reduce image misinterpretation, misdiagnosis, or unnecessary surgery. Missing records do not prove that testing or multidisciplinary review did not occur. AIP, autoimmune pancreatitis; IgG4-RD, immunoglobulin G4-related disease; MPD, main pancreatic duct; PDAC, pancreatic ductal adenocarcinoma.

## Data Availability

The datasets generated and/or analyzed during the current study are not publicly available because of institutional regulations and patient confidentiality requirements but may be available from the corresponding author upon reasonable request and with permission from the participating institution.

## Supplementary Materials

The following supporting information can be downloaded at: https://www.mdpi.com/article/10.3390/diagnostics16193129/s1, Figure S1: Cohort ascertainment and archived diagnostic-classification flow; Table S1: Aggregate JPS 2018 diagnostic evidence and data availability; Table S2: Retained histopathologic evidence for nine pathology-supported cases; Table S3: Retrievable chronology and evidence limitations in the six illustrative surgical cases. The supplement does not constitute an independent diagnostic-validation dataset.

## Abbreviations

ADC, apparent diffusion coefficient; AIP, autoimmune pancreatitis; CT, computed tomography; DWI, diffusion-weighted imaging; EUS, endoscopic ultrasound; FNA, fine-needle aspiration; FNB, fine-needle biopsy; IgG4, immunoglobulin G4; IgG4-RD, immunoglobulin G4-related disease; IgG4-SC, IgG4-related sclerosing cholangitis; JPS, Japan Pancreas Society; LPSP, lymphoplasmacytic sclerosing pancreatitis; MOSE, macroscopic on-site evaluation; MPD, main pancreatic duct; MRI, magnetic resonance imaging; MRCP, magnetic resonance cholangiopancreatography; PACS, picture archiving and communication system; PDAC, pancreatic ductal adenocarcinoma.
