# Supplementary material for: Extrapancreatic Findings and Retrospective Diagnostic Pitfalls in Type 1 Autoimmune Pancreatitis: A Descriptive CT and MRI Study
Source: Diagnostics (Basel). 2026 Sep 26;16(19):3129. doi: 10.3390/diagnostics16193129 (PMC13664398; doi:10.3390/diagnostics16193129)
Supplement: Supplementary file 1 [file diagnostics-16-03129-s001.zip › diagnostics-4562246-supplementary.pdf]

Supplementary Materials

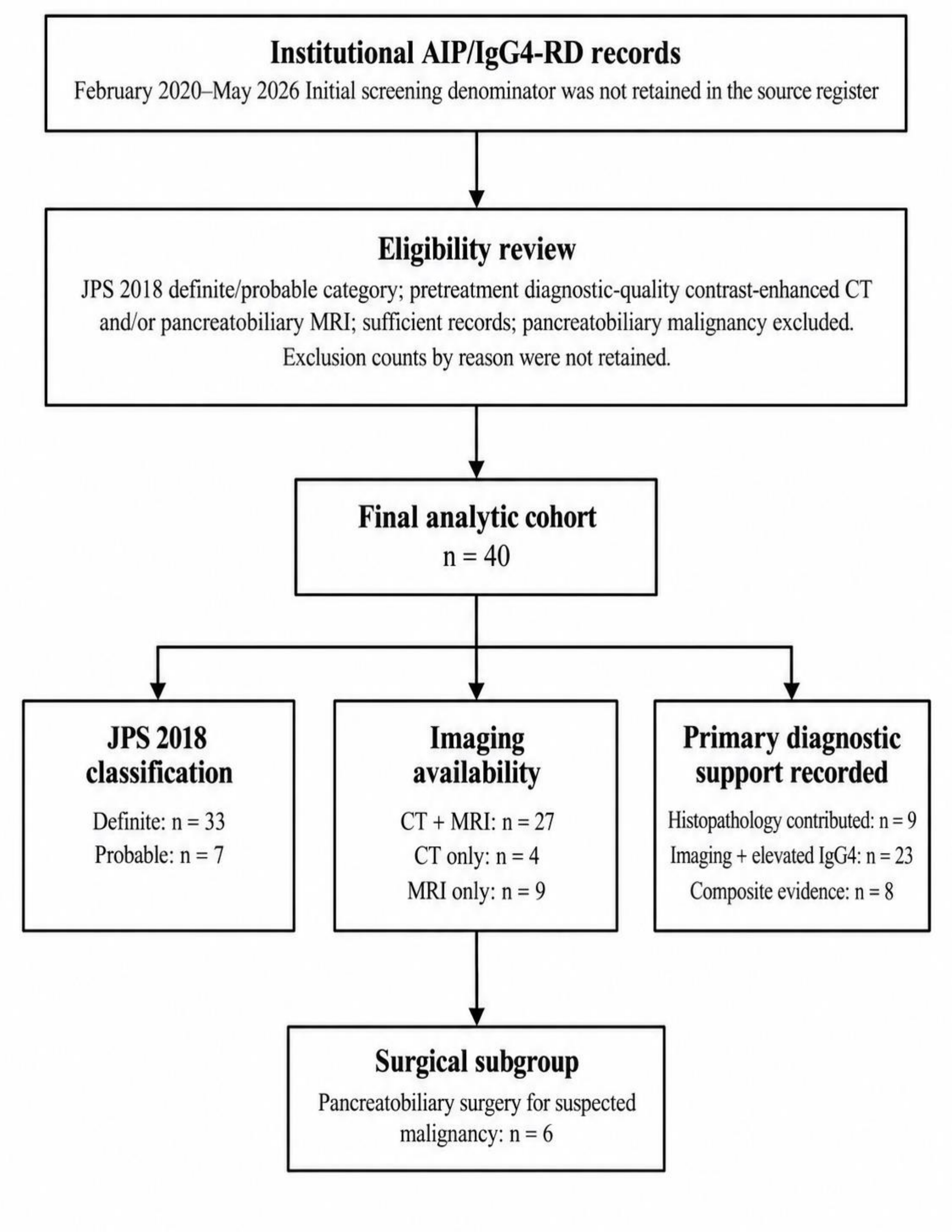

Figure S1. Cohort ascertainment and archived diagnostic-classification flow. The initial number screened and exclusions by reason were not retained and are not treated as zero. The diagnostic-support categories are archived summaries, not an independently reconstructed patient-level reference standard. The six operated patients are a nested illustrative case series; the post hoc analysis was descriptive and hypothesis-generating, without malignant controls.

Table S1. Aggregate JPS 2018 diagnostic evidence and data availability.

Domain	Retained classification / evidence	Count	Timing and criterion-level availability
Final JPS 2018 category	Archived definite classification	33	Final category retained; the patient-level combination of JPS domains was not retained in the analytic dataset.
Final JPS 2018 category	Archived probable classification	7	Final category retained; individual component-level adjudication could not be independently reconstructed.
Pancreatic morphology	Diffuse / non-diffuse	28 / 12	Morphology retained for every included patient.
Imaging availability	CT + MRI / CT only / MRI only	27 / 4 / 9	All 40 had at least one diagnostic-quality pretreatment contrast-enhanced examination.
Serum IgG4	Measured; >135 mg/dL; >280 mg/dL	32; 32; 26	Exact sampling dates relative to imaging and treatment were unavailable for 31/32; Case 06 was documented as preoperative.
Histopathology	Contributed to diagnostic adjudication	9	Archived pathology reports were used; central slide rereview and uniform JPS histology-component coding were not available.
Imaging + elevated IgG4	Principal recorded diagnostic support	23	The exact interval between IgG4 sampling and imaging was not retained.
Composite evidence	Imaging, IgG4, malignancy exclusion, histopathology and/or steroid response	8	The exact case-level combination and the number in which steroid response contributed were not separately coded.
Corticosteroid response	Potential supportive JPS domain after appropriate malignancy evaluation	Not separately retained	Index imaging preceded corticosteroid treatment. Case-level contribution, exact response timing, and verification of every diagnostic step were not retained.

CT, computed tomography; MRI, magnetic resonance imaging; JPS, Japan Pancreas Society.

Table S2. Retained histopathologic evidence for the nine pathology-supported cases.

Case	Specimen / source	Retained report-level findings	JPS histology components retained?	Scope of interpretation
Surgical case 01	Pancreatobiliary resection specimen	No malignancy; chronic pancreatitis/fibroinflammatory pancreatobiliary change was recorded in the archived summary as compatible with type 1 AIP/IgG4-RD.	Not consistently retrievable: IgG4-positive cells/HPF, storiform fibrosis, and obliterative phlebitis were not separately coded.	Histopathology contributed to multidisciplinary adjudication; no standalone JPS IVa/IVb claim was reconstructed.
Surgical case 02	Pancreatobiliary resection specimen	No malignancy; chronic pancreatitis/fibroinflammatory pancreatobiliary change was recorded in the archived summary as compatible with type 1 AIP/IgG4-RD.	Not consistently retrievable: IgG4-positive cells/HPF, storiform fibrosis, and obliterative phlebitis were not separately coded.	Histopathology contributed to multidisciplinary adjudication; no standalone JPS IVa/IVb claim was reconstructed.
Surgical case 03	Pancreatobiliary resection specimen	No malignancy; chronic pancreatitis/fibroinflammatory pancreatobiliary change was recorded in the archived summary as compatible with type 1 AIP/IgG4-RD.	Not consistently retrievable: IgG4-positive cells/HPF, storiform fibrosis, and obliterative phlebitis were not separately coded.	Histopathology contributed to multidisciplinary adjudication; no standalone JPS IVa/IVb claim was reconstructed.
Surgical case 04	Pancreatobiliary resection specimen	No malignancy; chronic pancreatitis/fibroinflammatory pancreatobiliary change was recorded in the archived summary as compatible with type 1 AIP/IgG4-RD.	Not consistently retrievable: IgG4-positive cells/HPF, storiform fibrosis, and obliterative phlebitis were not separately coded.	Histopathology contributed to multidisciplinary adjudication; no standalone JPS IVa/IVb claim was reconstructed.
Surgical case 05	Pancreatobiliary resection specimen	No malignancy; chronic pancreatitis/fibroinflammatory pancreatobiliary change was recorded in the archived summary as compatible with type 1 AIP/IgG4-RD.	Not consistently retrievable: IgG4-positive cells/HPF, storiform fibrosis, and obliterative phlebitis were not separately coded.	Histopathology contributed to multidisciplinary adjudication; no standalone JPS IVa/IVb claim was reconstructed.

Case	Specimen / source	Retained report-level findings	JPS histology components retained?	Scope of interpretation
Surgical case 06	Pancreatoduodenectomy specimen	No malignancy; chronic fibrosing inflammation of the common bile duct and chronic pancreatitis compatible with IgG4-RD; chronic active perforated gastroduodenal ulceration and Candida in necrotic tissue at the ulcer base.	IgG4-positive cells/HPF, storiform fibrosis, and obliterative phlebitis were not available in the retained extract.	Report-level fibroinflammatory evidence. Ulceration and Candida were postoperative findings, not preoperative evidence; their relationship to the EUS impression of invasion remains inferential.
Non-surgical pathology-supported Case A	Original pathology report referenced in archived adjudication; specimen type not retained in the analytic dataset.	Histopathology was recorded as contributing to the final AIP diagnosis; detailed report wording was not available in the retained analytic file.	Not retrievable.	Supportive evidence only; no criterion-level histologic classification was inferred.
Non-surgical pathology-supported Case B	Original pathology report referenced in archived adjudication; specimen type not retained in the analytic dataset.	Histopathology was recorded as contributing to the final AIP diagnosis; detailed report wording was not available in the retained analytic file.	Not retrievable.	Supportive evidence only; no criterion-level histologic classification was inferred.
Non-surgical pathology-supported Case C	Original pathology report referenced in archived adjudication; specimen type not retained in the analytic dataset.	Histopathology was recorded as contributing to the final AIP diagnosis; detailed report wording was not available in the retained analytic file.	Not retrievable.	Supportive evidence only; no criterion-level histologic classification was inferred.

HPF, high-power field; LPSP, lymphoplasmacytic sclerosing pancreatitis.

Table S3. Retrievable chronology and evidence limitations in the six illustrative surgical cases.

Case	Presentation	Retained imaging sequence and retrospective observations	Preoperative IgG4 / tissue evidence	Initial diagnosis	Operation	Postoperative evidence
01	Obstructive jaundice and abdominal pain	CT/MRI: focal pancreatic-head lesion with biliary obstruction. Speckled/progressive enhancement, capsule-like rim, and MPD abnormalities were identified retrospectively. Renal finding: see shared case-linkage note below.	IgG4 reported as not performed; no preoperative biopsy information retained.	Pancreatic-head tumor	Pancreatoduodenectomy	No malignancy; chronic fibroinflammatory pancreatitis recorded as compatible with type 1 AIP/IgG4-RD; component-level histology not retained.
02	Obstructive jaundice and abdominal pain	CT/MRI: focal pancreatic-head lesion with biliary obstruction. Compatible enhancement and ductal abnormalities were identified retrospectively. Renal finding: see shared case-linkage note below.	IgG4 reported as not performed; no preoperative biopsy information retained.	Pancreatic-head tumor	Pancreatoduodenectomy	No malignancy; chronic fibroinflammatory pancreatitis recorded as compatible with type 1 AIP/IgG4-RD; component-level histology not retained.
03	Obstructive jaundice and abdominal pain	MRI/CT: focal head–body lesion; arterial hypoenhancement, peripancreatic venous narrowing, MPD dilatation, and upstream atrophy created an appearance of locally advanced PDAC; later-phase enhancement and a capsule-like rim favored AIP.	No preoperative IgG4 result retained; no preoperative biopsy information retained.	Pancreatic head–body tumor	Extended pancreatoduodenectomy	No malignancy; chronic fibroinflammatory pancreatitis recorded as compatible with type 1 AIP/IgG4-RD; component-level histology not retained.

Case	Presentation	Retained imaging sequence and retrospective observations	Preoperative IgG4 / tissue evidence	Initial diagnosis	Operation	Postoperative evidence
04	Obstructive jaundice and abdominal pain	CT/MRI: diffuse pancreatic enlargement, intrinsic distal bile-duct mural/stricture abnormality, and bilateral renal lesions. The original working diagnosis emphasized the distal bile-duct lesion; a causal cognitive mechanism was not measured.	IgG4 reported as not performed; EUS information was not used as a decisive AIP criterion in the retained summary.	Distal common bile-duct tumor	Pancreatoduodenectomy	No malignancy; chronic pancreatobiliary fibroinflammatory change recorded as compatible with type 1 AIP/IgG4-RD; component-level histology not retained.
05	Obstructive jaundice and abdominal pain	Earlier imaging approximately six months before the later examination showed diffuse pancreatic enlargement and biliary abnormality. Later imaging showed atrophy and prominent hilar disease. Availability to the treating team at the decision time was not documented.	IgG4 reported as not performed; no preoperative biopsy information retained.	Klatskin tumor type IV	Biliary surgery with segmental hepatectomy	No malignancy; pancreatobiliary fibroinflammatory change recorded as compatible with IgG4-RD; component-level histology not retained.

Case	Presentation	Retained imaging sequence and retrospective observations	Preoperative IgG4 / tissue evidence	Initial diagnosis	Operation	Postoperative evidence
06	Obstructive jaundice, abdominal pain, and marked weight loss	Ultrasound: intra/extrahepatic biliary dilatation. MRI: distal common bile-duct wall thickening and diffuse/mild pancreatic enlargement without a focal mass. EUS: enlarged pancreas without focal mass; 2.4 × 2.5-cm distal common bile-duct lesion with suspected duodenal invasion and a duodenal ulcer. CT: speckled/head-predominant enhancement against a hypoenhancing body/tail.	IgG4 recorded as 897 mg/dL during preoperative work-up; assay interval, exact test dates, and decision-time communication were unavailable. Duodenal-ulcer mucosal biopsy: chronic inflammation, no malignant cells. No representative pancreatic/distal bile-duct core result was documented.	Pancreatic tumor or distal common bile-duct tumor invading the duodenum	Pancreatoduodenectomy	No malignancy; chronic fibrosing inflammation of the common bile duct and chronic pancreatitis compatible with IgG4-RD; chronic active perforated gastroduodenal ulceration and Candida in necrotic tissue.

Case-linkage note (Cases 01–02 / Figure 1): the retained narrative and prior Table S3 assigned the renal finding to Case 01, whereas the retained Figure 1 legend identified a 62-year-old woman, matching Case 02 in Table 4. No source-level verification resolving this discrepancy was supplied. The renal observation is therefore reported only as occurring in one of the two focal-head cases; it has not been reassigned to either patient. Figure 1 is retained as an unnumbered illustrative case, without the unverified demographic linkage. Cohort-level renal counts and the six-case demographic table remain unchanged.

Cases 04–06 retain the age, sex, morphology, and order in the English case table. Case 05 illustrates temporal change only; there is no cohort-wide prior-imaging denominator. None of these narratives establishes that a different interpretation would have safely prevented surgery.
